# Synthesis and Characterization of Hybrid Materials Consisting of *n*-octadecyltriethoxysilane by Using *n*-Hexadecylamine as Surfactant and Q^0^ and T^0^ Cross-Linkers

**DOI:** 10.3390/ijms13056279

**Published:** 2012-05-21

**Authors:** Ismail Warad, H Omar Abd-Elkader, Saud Al-Resayes, Ahmad Husein, Mohammed Al-Nuri, Ahmed Boshaala, Nabil Al-Zaqri, Taibi Ben Hadda

**Affiliations:** 1 Department of Chemistry, Science College, King Saud University, P.O. Box 2455, Riyadh 11451, Saudi Arabia; E-Mails: resayes@ksu.edu.sa (S.A.-R.); nabil_alzaqri@yahoo.com (N.A.-Z.); 2 Electron Microscope Unit, Zoology Department, College of Science, King Saud University, Riyadh 11451, Kingdom of Saudi Arabia; E-Mail: omabdelkader7@yahoo.com; 3 Electron Microscope & Thin Films Department, Physics Division, National Research Center, Dokki 12622, Cairo, Egypt; 4 Department of Chemistry, Science College, AN-Najah National University, P.O. Box 7, Nablus 00972, Palestine Territories; E-Mails: hamaydah2500@yahoo.com (A.H.); mabnuri@yahoo.com (M.A.-N.); 5 Chemistry Department, Faculty of Science, Benghazi University, P. O. Box 1308, Benghazi, 22385, Libya; E-Mail: ahmedboshaala@yahoo.co.uk; 6 Materials Chemistry Laboratory, Faculty of Sciences, University of Mohammed Premier, Oujda-60000, Morocco; E-Mail: taibi.ben.hadda@gmail.com

**Keywords:** sol-gel, solid state NMR, cross-linkers, stationary phases

## Abstract

Novel hybrid xerogel materials were synthesized by a sol-gel procedure. *n-*octadecyltriethoxysilane was co-condensed with and without different cross-linkers using **Q^0^** and **T^0^** mono-functionalized organosilanes in the presence of *n*-hexadecylamine with different hydroxyl silica functional groups at the surface. These polymer networks have shown new properties, for example, a high degree of cross-linking and hydrolysis. Two different synthesis steps were carried out: simple self-assembly followed by sol-gel transition and precipitation of homogenous sols. Due to the lack of solubility of these materials, the compositions of the new materials were determined by infrared spectroscopy, ^13^C and ^29^Si CP/MAS NMR spectroscopy and scanning electron microscopy.

## 1. Introduction

Alkyl stationary phases are widely used in liquid chromatography (LC). Much effort has been made to prepare and describe the chromatographic properties [1]. In this field, the application of stationary phases based on silica gel is very popular. For the successful employment of silica, it is of great importance that the silica beads show a narrow particle size distribution and spherical shape. Most HPLC separations are carried out under reversed phase (RP) conditions [2]. The surface modification of well-defined silica beads with T-silyl functionalized organic systems is the major route to create phases for reversed-phase liquid chromatography (RPLC) [3–5]. All the RP prepared by the different conventional methods (solution or surface polymerized modification and monomeric synthesis) mentioned above do not exhibit complete cross-linked **T** and **Q** units. For long-term stability of stationary phases it is very important to prepare one with a high degree of cross-linked ligands. This may be achieved using the sol-gel process.

Sol-gel processing of polysiloxanes to prepare hybrid inorganic-organic materials (HIOM) [6–9] is quite a promising technique in field of chromatography [10,11]. It has been widely investigated to prepare potential matrices for reporter molecules in chemistry sensors [12]. More recently, such materials have also received attention as catalyst supports, enzymes and catalytically active transitions metal complexes [13].

Recently, many investigations were extended to nanostructure mesoporous silicas which are based on inorganic-organic hybrid polymers [14–19]. These materials possess extremely high surface areas and accessible pores. Moreover, the pore size can be tuned with different sizes in the nanometer range by choosing template systems or with a co-solvent [15]. For the generation of stationary phases, T-functionalized silanes of the type F_n_-Si(OR)_3_ were sol-gel processed with and without co-condensation agents, which play an important role in controlling the density and the distance of the reactive centers; in general F_n_ represents either alkyl spacer alone or end by a metal complex [20]. These reactive centers are distributed across the entire carrier matrix and play an important role in catalysis [21–38].

In this work, hybrid materials were synthesized by a simple one-step self assembly followed by co-condensation of different silane cross-linkers with *n*-octadecyltrialkoxysilane as the chromatographic selector in the presence of *n*-hexadecylamine surfactant. Application of different alkoxysilane co-condensation agents, such as Si(OEt)_4_, Si(OMe)_4_ (**Q^0^**) and MeSi(OMe)_3_ (**T^0^**) during the sol-gel processes enable the building of polymer frameworks with different hydroxyl silica functional groups and new properties compared to surface modified silica gel.

## 2. Results and Discussion

### 2.1. Sol-Gel Processing

**Scheme 1:**
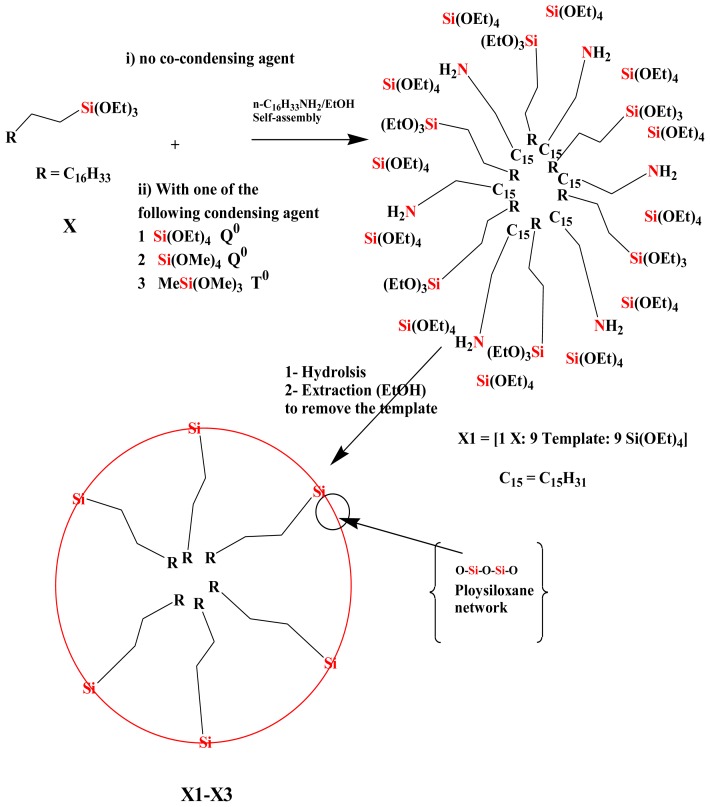
Synthesis of xerogels **X0**–**X3**: Self-assembly followed by sol-gel process at room temperature using several cross-linkers and the amine as template.

The properties of the sol-gel processed products strongly depend on the reaction conditions, such as type of solvent, temperature, time, catalysts, concentration of the monomers and type of the cross-linkers [9,10]. To ensure comparable results, uniform reaction conditions throughout hydrolysis and the sol-gel transition have been maintained. Alcohol is necessary to homogenize the reaction mixture. All poly-condensations were performed in a mixture of ethanol with a certain amount of water as catalyst for the sol-gel process (see Experimental section). Long alkyl chains commonly used for liquid chromatography were introduced by using the CH_3_(CH_2_)_17_Si(OEt)_3_, *n*-octadecyltriethoxysilane (**X**), in the presence of *n*-hexadecylamine as template, which concomitantly serves as material to allow mesoporous hybrid compounds. During the sol-gel process, a fixed ratio of **X**, template and co-condensation agents [1:9:9], respectively, were used. Several types of co-condensation agents used, such as Si(OEt)_4_ (TEOS), Si(OMe)_4_ (TMOS) and MeSi(OMe)_3_, are shown in Scheme 1.

After sol-gel processing, the amine was removed from the mixture by Soxhlet extraction with ethanol. Four xerogels (**X0**–**X3**) were collected and are illustrated in Table 1.

Two kinds of stationary phases were obtained: (i) xerogels **X0** were synthesized by co-condensation of **X** and template with zero concentration (**0**) of cross-linker, and (ii) xerogels **X1**–**X3** were synthesized by co-condensation of **X**, template with monofunctional **Q^0^** and **T^0^** cross-linkers such as Si(OEt)_4_, Si(OMe)_4_ and MeSi(OMe)_3_, respectively.

### 2.2. Solid-State NMR Spectroscopic Investigations

Due to cross-linking effects, the solubility of the polymeric materials **X0**–**X3** is rather limited. Therefore, solid-state NMR spectroscopy was used as a powerful technique for their characterization.

#### ^29^Si CP/MAS NMR spectroscopy

**Scheme 2:**
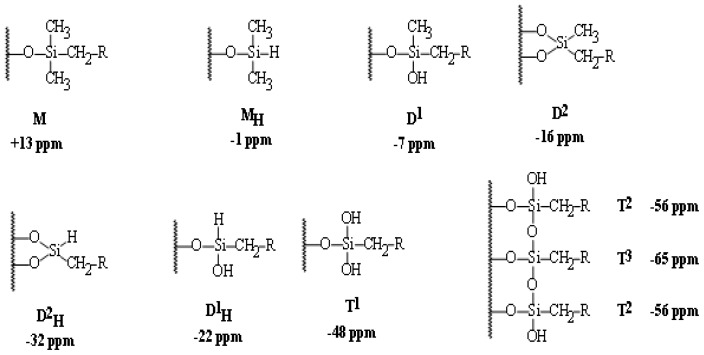

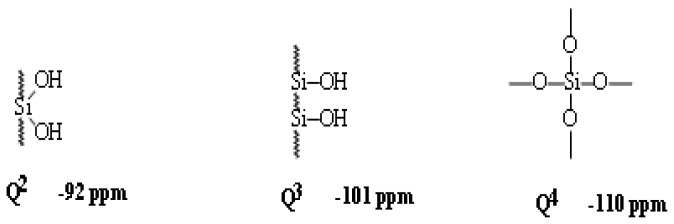
The universal ^29^Si chemical shifts, symbols and orders of silyl species.

Silane functionality and bonding chemistry can easily be determined by ^29^Si CP/MAS NMR spectroscopy. The universal chemical shift and symbols for these ^29^Si function groups are collected in Scheme 2.

The average chemical shifts for **T^2^** (δ = −58.8), and **T^3^** (δ = −65.1), **Q^3^** (δ = −101.9), **Q^4^** (δ = −109.5) species are significantly changed by the incorporation of the different types of co-condensation agents and are in agreement with values reported in the literature for comparable systems [37]. Since all silicon atoms are in direct proximity of protons the Hartmann–Hahn [29–33] match could efficiently be achieved.

The signal assignment of silyl fragments can be summarized as follows: a higher degree of cross-linking of silicon species and/or an increase of oxygen neighbors leads to an upfield shift in NMR spectra. Difunctional species (Dn) appear in the region of −7 to −20 ppm, tri-functional species (Tn) from −49 to −66 ppm, and signals from the native silica (Qn) from −91 to −110 ppm. The high condensation degrees which were obtained through the sol-gel processes and monitored by 29Si NMR is mainly resonated to the employment of **T^2^**, **T^3^**, **Q^3^** and **Q^4^** as cross-linkers [28–35].

**Figure 1:**
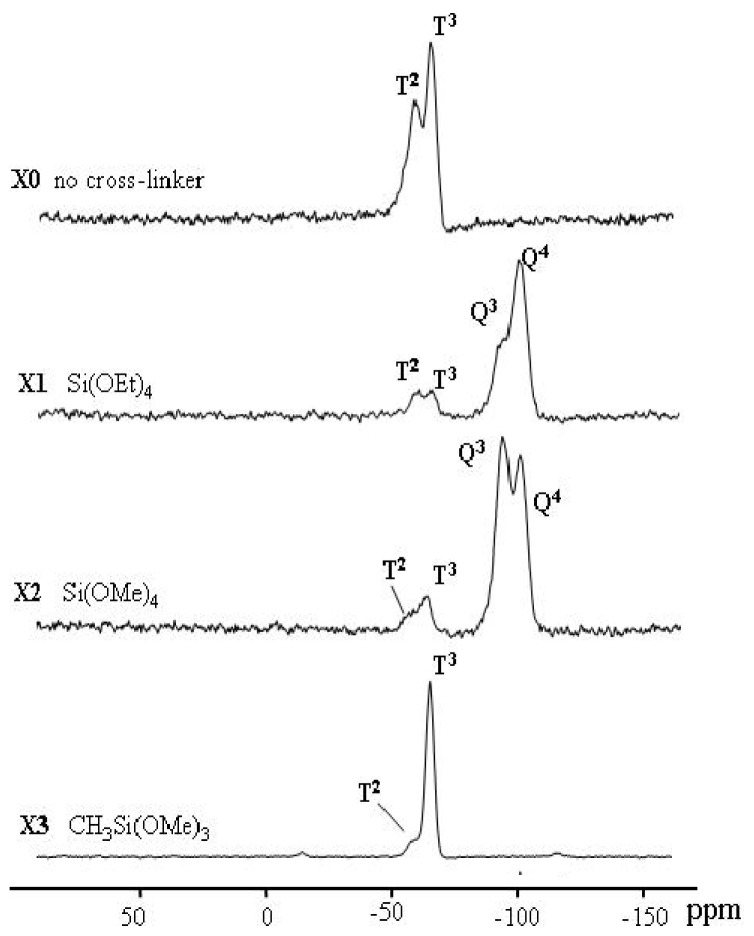
^29^Si CP/MAS NMR spectra of **X0**–**X3** materials which were prepared by using Si(OEt)_4_, Si(OMe)_4_ and MeSi(OMe)_3_ as cross-linkers.

All the spectra of xerogels (**X0–X3**) show signals for substructures corresponding to **T** and **Q** functions only, the **D** or **M** silyl fragment has not been observed, as seen in Figure 1. **X0**–**X3** showed **T** silyl fragment only in general 3 order predominated over 2, which supports the full cross-linked organosiloxane species, demonstrating the incorporation of the function groups within the framework walls of the mesostructures. **T^2^** silyl fragment indicted that one Si-OH function group of the sector or cross-linkers MeSi(OMe)_3_ was not incorporated in the sol-gel process. **X0** and **X3** revealed only T silyl fragment, and this was expected since no **Q** co-condensation agents were used. **X1** and **X2** showed both **T** and **Q** with 2 and 3 order, because both the sector **T** silyl fragment and **Q** silyl fragment of the co-condensation agents were investigated in the sol-gel process; **Q** predominated over **T** due to the concentrations used [9:1] respectively. The respective NMR spectra of the different materials’ phases and the native silica are shown in Figure 1.

### 2.3. ^13^C CP/MAS NMR Spectroscopy

**Figure 2:**
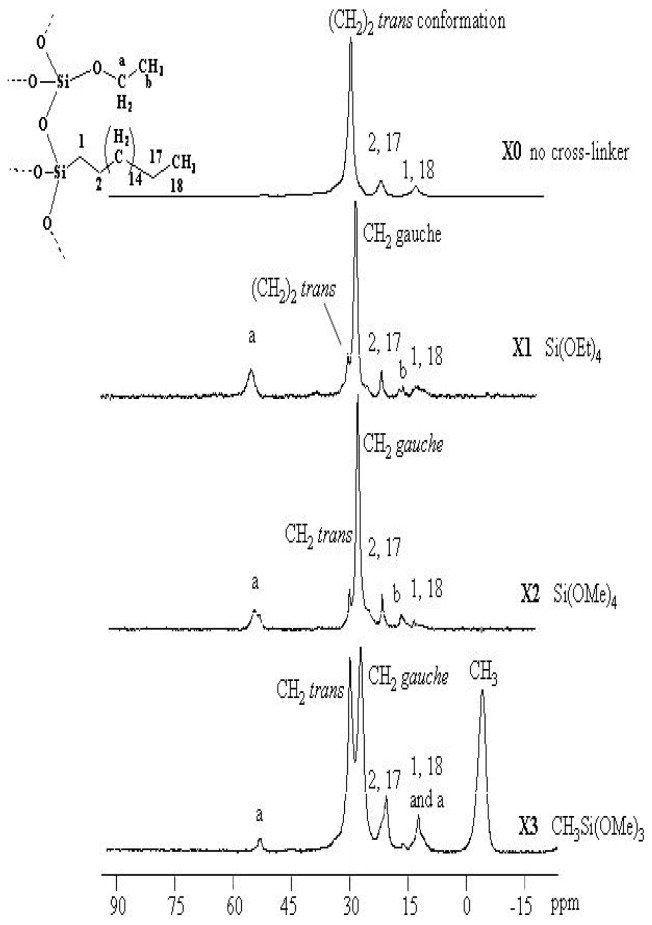
^13^C CP/MAS NMR spectra of **X0-X3** materials.

More detailed information on bonded phase architecture was obtained from the ^13^C CP/MAS NMR spectra of the supported matrices **X0**–**X3**, and the corresponding signal assignments are summarized in Figure 2. Characteristic peaks at approximately δ = 14.0 ppm are assigned to the carbon atom of CH_3_ and the carbon atom of the silicon adjacent methyl groups (SiCH_2_) in the Si–O–Si substructure δ = 22.8 ppm is assigned to CH_2_CH_3_ and SiCH_2_*C*H_2_ are significant for all the stationary phases. Weak signals of ^13^C were assigned to residual Si–OR (R = Me or Et) functionalities at around δ = 17.0 and 57.9 ppm, attributed to non-hydrolyzed EtO (**X1**) or MeO (**X2** and **X3**) silica functional groups, which point to a high degree of hydrolysis compared to the literature [7,9]. δ = −3.6 ppm is assigned to SiMe in case of using MeSi(OMe)_3_. The NMR investigation of such stationary bonded phases exhibits two signals for the main chain methylene carbons at 30.0 and 32.2 ppm (*gauche*/*trans* conformations, respectively) as shown in Figure 2 [5].

The samples **X1**, **X2** and **X3** show two peaks related to the main chain (CH_2_)_14_ at 30.0 and 32.0 ppm which represent two different conformations of the alkyl chain. The signal at 32.8 ppm characterizes *trans* conformations, and the signal at 30.0 ppm reveals the existence of *gauche* conformations. The effect of different motilities is only visible in the ^13^C CP/MAS NMR spectra of the xerogels containing alkyl chains with 18 carbon atoms at least; the alkyl chain order is found to increase with increasing chain length from C_18_ to C_34_ [36].

**Scheme 3:**
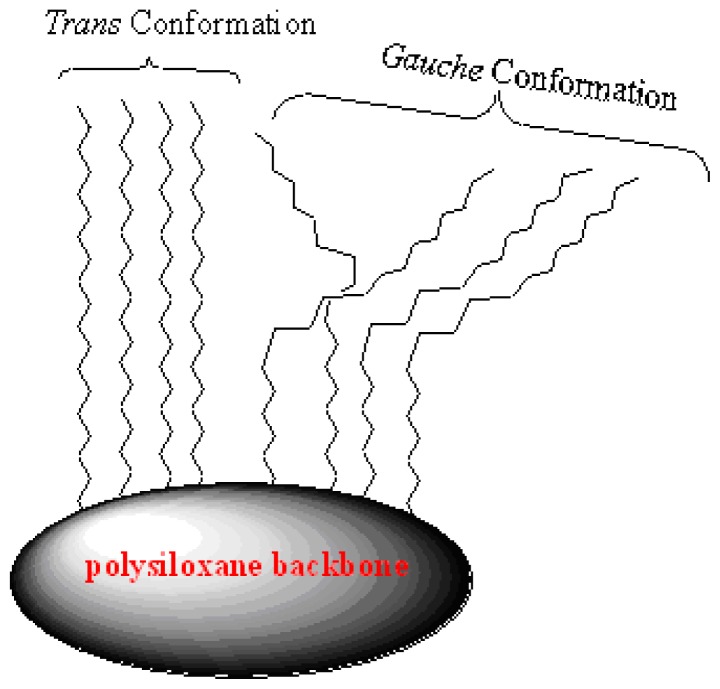
Schematic illustration of *trans* and *gauche* alkyl chain arrangements.

The NMR investigation of C_18_ bonded phases here exhibits only one signal for the main chain: methylene carbons at 32.8 belong to the *trans* conformation when no co-condensation agent was investigated (**X0**). When TESO or TMSO (**Q^0^**) was introduced to the sol-gel, the presence of the Me-Si function group in the cross-linker plays a key role in the conformation; the *trans* conformation was encouraged. The main reason of such a phenomenon is not yet clear, it could be related to the un-hydrolysis Me-Si steric factor of remaining Me on the **X3** polysiloxane surface affecting the C_18_ chain not to bend away to form a *gauche* conformation, thus enhancing the C–H Wander Val forces to form sort of stabilizing straight line chains as in Scheme 3. This proposal is compatible with the decrease in the *trans* conformation at the expense of the alternative *gauche* conformation increasing upon increasing the temperature [9,28–30].

Here we report a new method at room temperature to control the *gauche*/*trans* conformation ratio of the alkyl chains in the stationary phase by using fixed concentrations of several type of **T^0^** and **Q^0^** cross-linkers.

### 2.4. IR Investigations

**Figure 3:**
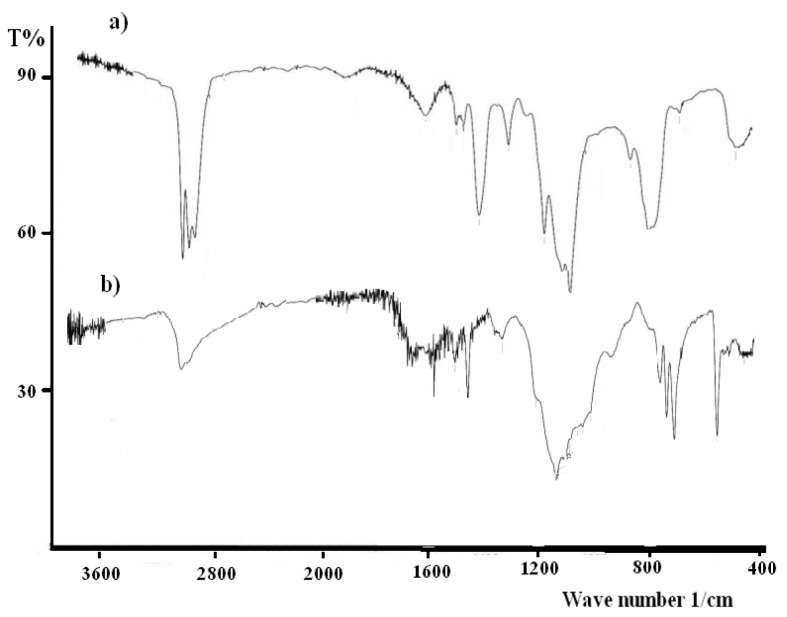
Infra-red spectra (**a** and **b**) of **X** and **X3**, before and after sol-gel, respectively.

The IR spectra of the desired materials, **X0**, **X1**, **X2** and **X3** in particular, show several peaks that are attributed to stretching and bending. The broad intensive stretching vibrations at 2980–2840 cm^−1^ and bending vibrations at 1150–950 cm^−1^ belonging to (*v*_CH_) of the SiCH_2_(CH_2_)_16_CH_3_ functional groups were the main IR active functional groups. A typical example of the IR behavior of **X3** s illustrated in Figure 3b.

In order to confirm the full sol-gel reaction, the structural vibration behaviors of these compounds against the infrared spectra of **X** as the starting material and **X3** as xerogel before and after the sol-gel processes were investigated and compared, such as the typical examples in Figure 3.

The broad intensive stretching vibrations at 2980–2840 cm^−1^ of (v_CH_) belong to the SiOCH_2_CH_3_ functional groups of **X** starting material as in Figure 3a totally disappeared after the sol-gel process to prepare the complex **X3** as seen in Figure 3b, which strongly confirms the completeness of the sol-gel process formation.

### 2.5. Surface Structure of the Materials

The surface area data (BET) of such materials determined by the sol-gel procedure was found to equal 1000–1500 m^2^/g, 0.5–1.3 cm^3^/g pore volume, and 13–20 Ǻ pore size [37].

**Figure 4:**
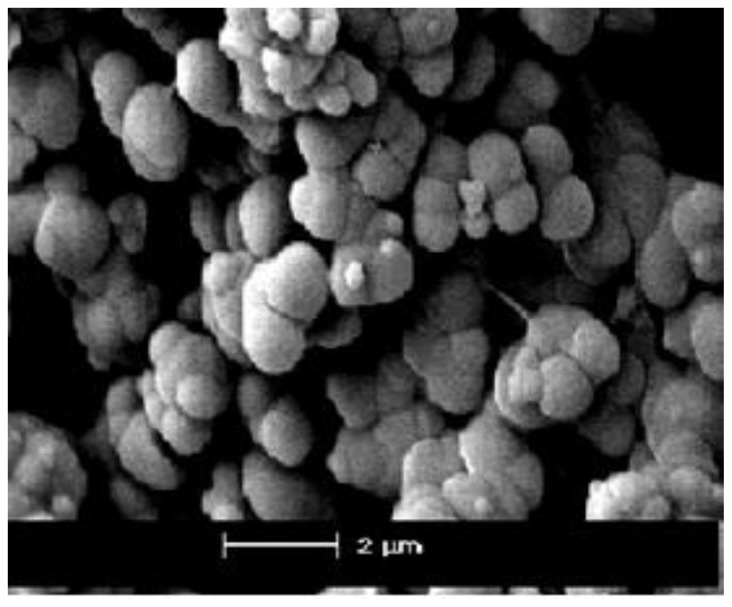
Scanning electron micrograph (SEM) of **X2**.

SEM micrographs of the **X_2_** and **X_3_** powder are given in Figure 4. Morphological features of the samples show the difference between spherical shapes, colloidal and porous structure and irregular particles mainly with the size 0.5–2 μm. All powders consisted of hard sub micrometer agglomerates, which are composed of fine crystallites. Generally, these agglomerate particles are hard to break down even with a long ultra-sonication time.

## 3. Experimental

### 3.1. General Methods

All reactions were carried out in an inert atmosphere (argon) by using standard high vacuum and Schlenk-line techniques unless otherwise noted. *n*-octadecyltriethoxysilane was purchased from ABCR GmbH, Germany. Prior to use, *n*-hexane and Et_2_O were distilled from both LiAlH_4_ and sodium/benzophenone, respectively. All other chemicals were obtained from Merck KGaA Darmstadt, Germany, and were used without further purification.

### 3.2. Method of Characterization

^13^C CP/MAS NMR spectra were recorded on a Bruker ASX 300 spectrometer (Bruker GmbH, Rheinstetten, Germany) at a spinning rate of 4000 Hz with 7 mm double bearing rotors of ZrO_2_ and proton of 90° pulse length and 7 μs was used. The contact time and delay time were 3 ms and 3 s, respectively with the line broadening of 30 Hz.

^29^Si CP/MAS NMR spectra were also collected on a Bruker ASX 300 NMR spectrometer. Representative samples of 200–250 mg were spun at 3500 Hz using 7 mm double bearing ZrO_2_ rotors. The spectra were obtained with a cross-polarization contact time of 5 ms. The pulse interval time was 1 s. Typically, 1.5 k FIDs with an acquisition time of 30 ms were accumulated in 1 kb data points and zero-filling to 8 kb prior to Fourier transformation. Line broadening of 30 Hz and respective spectral width were found for all spectra at about 20 kHz.

Scanning electron microscopy was performed on a JEOL JSM-6380 LA scanning electron microscope (SEM).

### 3.3. General Procedure for the Sol-Gel Processes

2.2 × 10^−2^ mmol of *n*-hexadecylamine (template) in 50 mL of absolute ethanol was completely dissolved. The addition of 2.4 × 10^−3^ mmol of *n*-octadecyltriethoxysilane (selector) to the template solution and stirring for 3 h is an important step for self-assembly. 2.2 × 10^−2^ mmol of the corresponding cross-linker was added and stirred for 10 h, at 35 °C with a molar substrate [1:9:9] [Selector:Template:Cross-linker] respectively. An excess (5 g) of distilled water was added drop wise to a stirred solution. This mixture was stirred for 24 h at room temperature until a gel was formed, then the solvent was removed by reducing the pressure. For the removal of *n*-hexadecylamine the crude xerogels were placed in a Soxhlet extractor containing 300 mL ethanol and the mixture was refluxed for 3 days. Subsequently the gels were washed three times with *n*-hexane and ether (100 mL) respectively, and dried under vacuum for 12 h.

#### Polysiloxanyloctane (X0)

A mixture of 1 g (2.4 × 10^−3^ mmol) of *n*-octyltrimethoxysilane and 5.2 g (2.2 × 10^−2^ mmol) *n*-hexadecylamine were sol-gel processed in ethanol and water to yield a colorless swollen gel. After purification and drying 0.6 g of white powder was formed. ^13^C CP/MAS NMR δ = 14.0 (SiCH_2_ and CH_3_), 22.8 (SiCH_2_*C*H_2_ and *C*H_2_CH_3_), 32.8 [(CH_2_)_14_ *trans* conformation]. ^29^Si CP/MAS NMR δ = −57.8 (**T^2^**), −67.1 (**T^3^**).

#### Polysiloxanyloctane (X1)

A mixture of 1 g (2.4 × 10^−3^ mmol) of *n*-octyltrimethoxysilane, 5.2 g (2.2 × 10^−2^ mmol) *n*-hexadecylamine and 5.5 g (2.2 × 10^−2^ mmol) of TEOS were sol-gel processed in ethanol and water to yield a colorless swollen gel. After purification and drying 5.2 g of white powder was formed. ^13^C CP/MAS NMR: δ = 13.2 (SiCH_2_ and CH_3_), 16.6 (OCH_2_*C*H_3_), 22.8 (SiCH_2_*C*H_2_ and *C*H_2_CH_3_), 29.9 [(CH_2_)_14_ *gauche* conformation], 32.0 [(CH_2_)_14_ *trans* conformation], 59.3 (OCH_2_). ^29^Si CP/MAS NMR: δ= −56.2 (**T^2^**), −66.8 (**T^3^**), −101.9 (**Q^3^**), −109.5 (**Q^4^**).

#### Polysiloxanyloctane (X2)

A mixture of 1 g (2.4 × 10^−3^ mmol) of *n*-octyltrimethoxysilane, 5.2 g (2.2 × 10^−2^ mmol) *n*-hexadecylamine and 5.5 g (2.2 × 10^−2^ mmol) of TMOS were sol-gel processed in ethanol and water to yield a colorless swollen gel. After purification and drying 4.2 g of white powder was formed. ^13^C CP/MAS NMR: δ = 13.2 (SiCH_2_ and CH_3_), 16.6 (OCH_2_*C*H_3_), 22.8 (SiCH_2_*C*H_2_ and *C*H_2_CH_3_), 29.9 [(CH_2_)_14_ *gauche* conformation], 32.0 [(CH_2_)_14_ *trans* conformation], 57.8 (OCH_2_).^29^Si CP/MAS NMR: δ = −57.2 (T^2^), −67.8 (T^3^), −101.5 (Q^3^), −109.9 (Q^4^).

#### Polysiloxanyldodecane (X3)

A mixture of 1g (2.4 × 10^−3^ mmol) of *n*-octyltrimethoxysilane, 5.2 g (2.2 × 10^−2^ mmol) *n*-hexadecylamine and 5.2 g (2.2 × 10^−2^ mmol) of MeSi(OMe)_3_ were sol-gel processed in ethanol and water to yield a colorless swollen gel. After purification and drying 4.5 g of white powder was formed. ^13^C CP/MAS NMR: δ = −3.6 (CH_3_Si), 14.0 (SiCH_2_ and CH_3_), 17.2 (OCH_2_*C*H_3_), 22.8 (SiCH_2_*C*H_2_ and *C*H_2_CH_3_), 29.9 [(CH_2_)_14_ *gauche* conformation], 32.8 9 [(CH_2_)_14_ *trans* conformation], 57.4 (OCH_2_). ^29^Si CP/MAS NMR: δ = −57.2 (T^2^), −67.8 (T^3^).

## 4. Conclusion

The sol-gel process offers new hybrid materials **X0**, **X1**, **X2** and **X3**. A suitable pathway is the sol-gel processing of *n*-alkyl like *n*-octadecyltriethoxysilane with an aliphatic amine like *n*-hexadecylamine as template molecules and different cross-linkers such as Si(OEt)_4_, Si(OMe)_4_ (**Q^0^**) and MeSi(OMe)_3_ (**T^0^**) carried out in ethanol at room temperature. The structure of all xerogels (**X0**–**X3**) were determined by solid state ^13^C and ^29^Si NMR spectroscopy, infrared spectroscopy and SEM. Additionally, the mobility of the alkyl chains and the dynamic behavior of the polymer matrix depended strongly on the type of cross-linkers. At room temperature, pure *trans* was observed without cross-linker while trace amount of *trans* was detected when **Q^0^** cross-linkers were introduced. Both *trans* and *gauche* conformations in equal amounts were recorded when **T^0^** cross-linkers were used.

## Figures and Tables

**Table 1:** Sol-gel processes, yields and labeling of the materials.

No.	Xerogel	Cross-Linkers Types	Yield %	Silyl Fragments
1	**X0**	-	60.0	**T^2^** and **T^3^**

2	**X1**	Si(OEt)_4_	77. 6	**T^2^**, **T^3^**, **Q^3^** and **Q^4^**
3	**X2**	Si(OMe)_4_	64.5	**T^2^**, **T^3^**, **Q^3^** and **Q^4^**
4	**X3**	MeSi(OMe)_3_	72.5	**T^2^** and **T^3^**
